# Cancer incidence rates and trends among children and adolescents in Piedmont, 1967–2011

**DOI:** 10.1371/journal.pone.0181805

**Published:** 2017-07-24

**Authors:** Elena Isaevska, Milena Manasievska, Daniela Alessi, Maria Luisa Mosso, Corrado Magnani, Carlotta Sacerdote, Guido Pastore, Franca Fagioli, Franco Merletti, Milena Maule

**Affiliations:** 1 Childhood Cancer Registry of Piedmont, Cancer Epidemiology Unit, Department of Medical Sciences, University of Turin, Turin, Italy; 2 Department of Translational Medicine, Unit of Medical Statistics and Cancer Epidemiology, CPO Piemonte and University of Piemonte Orientale, Novara, Italy; 3 Pediatric Onco-Haematology, Stem Cell Transplantation and Cellular Therapy Division, Regina Margherita Children's Hospital, Turin, Italy; Universita degli Studi di Napoli Federico II, ITALY

## Abstract

In the past, increases in childhood cancer incidence were reported in Europe and North America. The aim of this study is to show updated patterns of temporal behavior using data of the Childhood Cancer Registry of Piedmont (CCRP), a region with approximately 4.5 million inhabitants in North-West Italy. CCRP has been recording incident cases in children (0–14 years) since 1967 and in adolescents (15–19) since 2000. Time trends were estimated as annual percent change (APC) over the 1976–2011 period for children, and over 2000–2011 for both children and adolescents. CCRP registered 5020 incident cases from 1967 to 2011. Incidence rates were 157 per million person-years for children (1967–2011) and 282 for adolescents (2000–2011). From 1976–2011, increasing trends were observed in children for all neoplasms (APC 1.1, 95%CI: 0.8; 1.5) and for both embryonal and non-embryonal tumors: 1.1%, (0.5; 1.6) and 1.2%, (0.7; 1.6), respectively. Increases were observed in several tumor types, including leukemia, lymphoma, central nervous system tumors and neuroblastoma. In 2000–2011, incidence rates showed mostly non statistically significant variations and large variability. The observation of trends over a long period shows that the incidence of most tumors has increased, and this is only partially explained by diagnostic changes. Large rate variability hampers interpretation of trend patterns in short periods. Given that no satisfying explanation for the increases observed in the past was ever found, efforts must be made to understand and interpret this peculiar and still ununderstood pattern of childhood cancer incidence.

## Introduction

In the last decades of the previous millennium, strong and unexplained increases in the incidence of childhood tumors were observed in Europe and USA [1–3]. In Italy, the AIRTUM (the Italian Association of Cancer Registries) working group in its 2008 report [4] showed alarming increases in incidence rates, even emphasized by media, whereas its edition from 2012 [5] showed that incidence rates seemed to be no longer increasing. The Childhood Cancer Registry of Piedmont (CCRP) reported increasing incidence trends during the period 1967–2001 [6], specifically for leukemias, central nervous system (CNS) tumors and neuroblastomas. Taking advantage of the very long series of childhood cancer cases records of the CCRP, our aim is to show updated patterns of temporal behavior of childhood cancer incidence. We have calculated incidence rates for the whole period of CCRP registration, from 1967 to 2011, and estimated time trends in two periods: from 1976 to 2011, a long time interval covering 36 years of registration of childhood cancer cases; and from 2000–2011, and a short time interval covering the last available 12 years of registration of both childhood and adolescents cancer cases.

## Methods

### The Childhood Cancer Registry of Piedmont

Data were obtained from the CCRP, the oldest and largest pediatric population-based cancer Registry active in Italy. The registry has been recording cancer cases in children (0–14 years) since 1965 and in adolescents (15–19 years) since 2000. The first 2 years of registration were used to identify prevalent cases whereas data from 1967 onwards have been used to estimate the incidence. Before avaliability of the hospital admission and discharge files (from mid 90s), cases were searched through manual perusal of hospital registries and clinical records. From then on, cases are identified from the Hospital Admission and Discharge files, including hospital stays of all Piedmont residents in all Italian hospitals. The potentially relevant discharge diagnoses are then selected, and their complete clinical documentation is requested and examined by an experienced pediatrician in order to identify incident cases. Further case ascertainment is obtained from the databases of pathology departments of Piedmont hospitals, records for reimbursements for cancer treatments administered abroad, the Turin Cancer Registry and the database of the Italian Association of Pediatric Hematology and Oncology. Only CCRP authorized personnel can access individual data. Data are anonymised before statistical analysis.

CCRP data quality indicators by period of registration are shown in Table 1.

**Table 1:** Childhood Cancer Registry of Piedmont 1967–2011: Data quality indicators by calendar period. Total number of cases per period (N), cases (number and percentage) identified through death certificate only (DCO), microscopically verified (MV) cases, cases with ill-defined or unspecified site (ICD-10: C26, C39, C48, C76, C80), cases based on imaging only, by calendar period of registration.

Period of registration	N	DCO		MV		Ill-defined site		Imaging	
		*n*	*%*	*n*	*%*	*n*	*%*	*n*	*%*
1967–1976	874	55	6.29	717	82.04	27	3.09	97	11.10
1977–1986	1078	1	0.09	994	92.21	5	0.46	78	7.24
1987–1996	921	0	0.00	887	96.31	14	1.52	32	3.47
1997–2006	1347	0	0.00	1304	96.81	24	1.78	43	3.19
2007–2011	800	0	0.00	764	95.50	3	0.38	36	4.50
Total	5020	56	1.12	4666	92.95	73	1.45	286	5.70

Cases are included in the CCRP only after positive confirmation of age and residence in Piedmont at diagnosis, verified at the residence Town Office Registrar. All diagnoses are coded using the International Classification of Diseases for Oncology, Third Edition (ICD-O-3) [7] classification and then grouped into the 12 main diagnostic groups of the International Classification of Childhood Cancer, Third Edition (ICCC-3) [8]. In the analyses we have included all malignant tumors, and malignant and benign tumors of the CNS. In addition to the ICCC groups, we analyzed embryonal tumors as a separate category. There is no widely accepted definition for embryonal tumors and different authors have used different definitions [5,9–12]. In the absence of a unique classifying system, we have grouped all the tumor types explicitly described in the World Health Organization (WHO)/ International Agency for Research on Cancer (IARC) Classification of Tumors series (“WHO blue books”) [13–21] as possibly originating from the embryonic tissue.

The list of embryonal tumors includes the following ICD-O-3 morphological codes: 8902, 8910, 8912, 8959, 8960, 8963, 8970, 8972, 8973, 9110, 9270, 9290, 9310, 9330, 9342, 9362, 9370, 9371, 9372, 9392, 9470, 9471, 9472, 9473, 9474, 9480, 9490, 9500, 9501, 9502, 9508, 9510, 9511, 9512, 9513, 9520, 9521 and 9522.

From 1970 to 1975 the registration was limited to the Province of Torino.

### Statistical analyses

Crude incidence rates were calculated as the number of new cases per million person-years for the 12 main ICCC diagnostic groups as well as for the most important subgroups, by age class. The analysis of incidence trends was restricted to the period 1976–2011 to include data from the whole region of Piedmont (registration was limited to the Province of Torino in the period 1970–1975) and to avoid major diagnostic changes in the diagnosis and classification of leukemia. Time trends were estimated also separately for the last available 12 years of observation (2000–2011), when the analysis is possible in both children and adolescents.

With regard to CNS tumors, we started the time trend analysis from 1982 in order to avoid biases due to improvements in diagnostics (such as the use of CT scans at the beginning of the 1980’s). Due to the heterogeneity of behavior, we also estimated time trends for all histologically verified CNS tumors by WHO grading [13]. The idea is that more sensitive diagnostic techniques might have artificially increased the incidence of slow growing tumors with lower grading but have only marginally affected the incidence of the aggressive malignant tumors with rapid growth and higher grading. Exclusion of cases with no histological verification ensures that if an increased trend is observed, this cannot be ascribed to an improved capability of identifying benign lesions that are not surgically removed.

For neuroblastomas we conducted a sensitivity analysis starting the time trend analysis from 1990, to account for the introduction of ultrasound techniques for the early detection of newborn hip dysplasia.

The trend in incidence rates over time was estimated as the annual percent change (APC) of the incidence rates, using the Joinpoint Regression Program [22]. For the calculation of APC in the 0–14 age group we used age standardized rates and their standard errors, using the world standard population (WHO 2000–2025). For the calculation of APC in the <1 and 15–19 age groups, we fitted log-linear regression models to data assuming a Poisson distribution.

## Results

**Fig 1:**
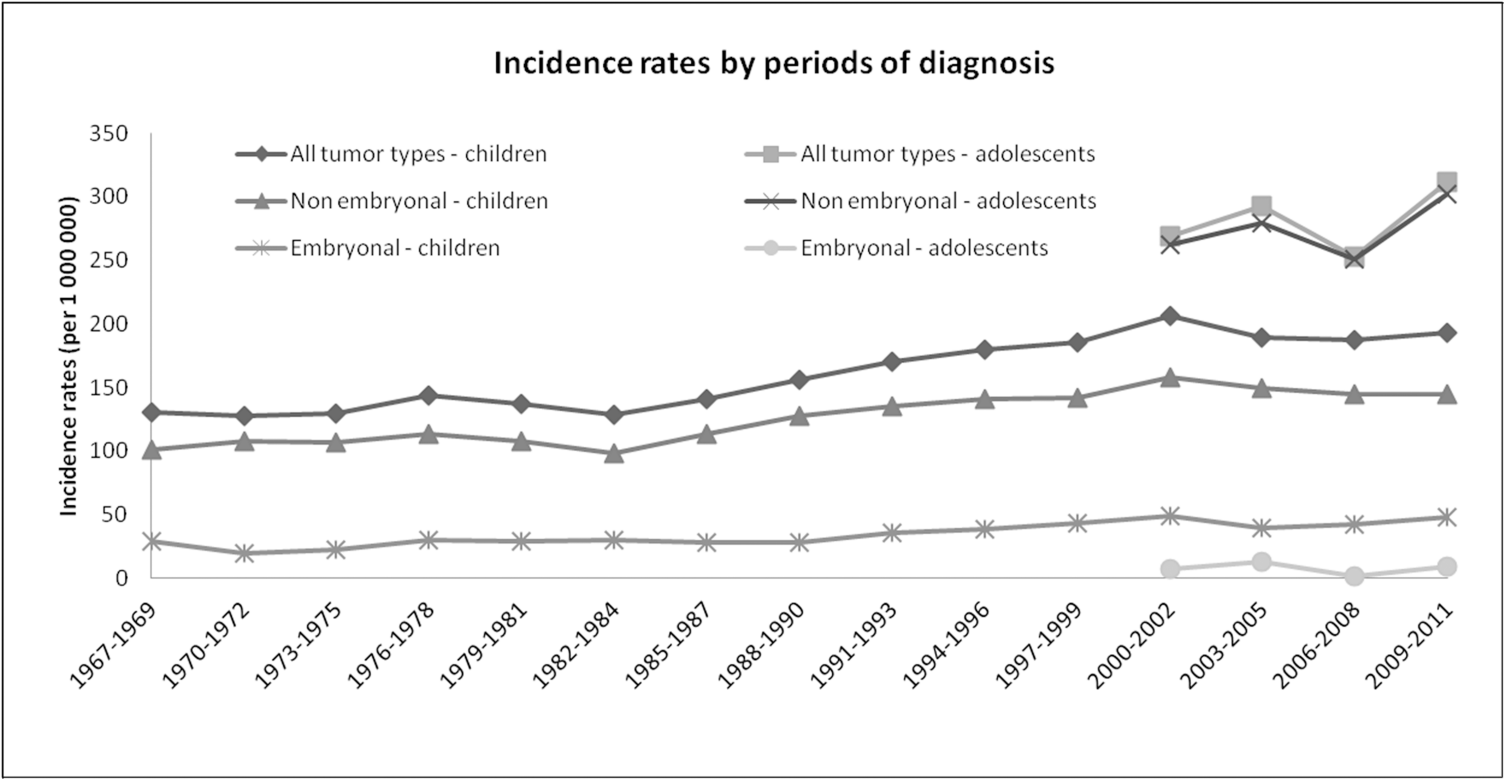
Childhood Cancer Registry of Piedmont 1967–2011: Incidence rates in children and adolescents by 3 year periods. (Data for adolescents available from 2000).

**Fig 2:**
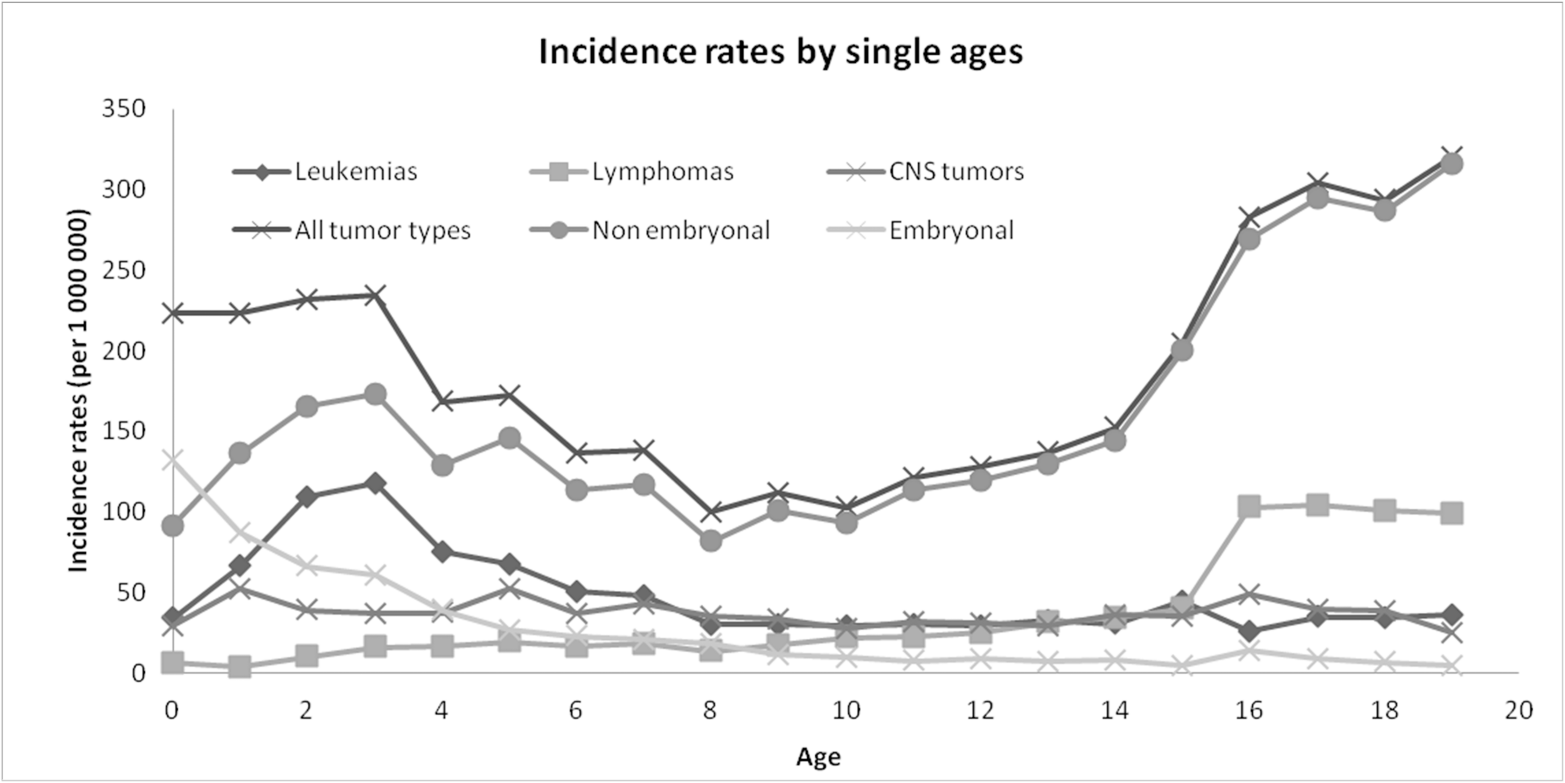
Childhood Cancer Registry of Piedmont 1967–2011: Incidence rates in children and adolescents by single year of age. (Data for adolescents available from 2000).

In 1967–2011, CCRP registered 5020 incident cases. The incidence rate of all tumor types for children (0–14 years) was 156.9 (151.3–161.6) (Table 2). In 2000–2011, the incidence rate for adolescents (15–19 years) was 281.7 (259.8–305.0) (Table 2). There was a statistically significant increase in incidence rates of all tumor types in children for the overall period starting 1976–2011, with APC 1.1% (0.8; 1.5), as well as of both embryonal and non-embryonal tumors: APC 1.1% (0.5; 1,6) and 1.2% (0.7; 1.6), respectively (Table 3, Fig 1). Embryonal tumors incidence peaks in the 0 age group and declines with age (Table 2, Fig 2), and their trend in the period 1976–2011 is mostly due to the increase in the first year of life, APC 1.5% (0.4; 2.7).

**Table 2:** Childhood Cancer Registry of Piedmont 1967–2011: Incidence rates by age groups. Number of cases (n), incidence rates per million per year (rate) and male-to-female ratio (M/F).

Childhood cancer registry of Piedmont 1967–2011^1^ International Classification for Childhood Cancer, Third edition	0 yrs		1–4 yrs		5–9 yrs		10–14 yrs		0–14 yrs			15–19 yrs		0–19 yrs		
	*n*	*rate*	*n*	*rate*	*n*	*rate*	*n*	*rate*	*n*	*rate*	*M/F*	*n*	*rate*	*n*	*rate*	*M/F*
I Leukemias	58	34.0	654	92.2	425	45.1	302	30.5	1439	51.2	1.2	76	35.2	1515	50.0	1.2
(a) Lymphoid leukemia	24	14.1	538	75.9	306	32.5	181	18.3	1049	37.3	1.2	36	16.7	1085	35.8	1.2
(b) Acute myeloid leukemia	17	10.0	51	7.2	58	6.2	73	7.4	199	7.1	1.2	27	12.5	226	7.5	1.1
(c) Chronic myeloproliferative diseases	3	1.8	4	0.6	14	1.5	15	1.5	36	1.3	1.1	8	3.7	44	1.5	0.9
(d) Myelodysplastic syndrome	2	1.2	3	0.4	2	0.2	2	0.2	9	0.3	0.5	4	1.9	13	0.4	0.9
(e) Unspecified and other leukemias	12	7.0	58	8.2	45	4.8	31	3.1	146	5.2	1.1	1	0.5	147	4.9	1.1
II Lymphomas	11	6.5	84	11.8	161	17.1	270	27.3	526	18.7	2.5	194	89.7	720	23.8	2.0
(a) Hodgkin lymphoma	2	1.2	18	2.5	54	5.7	141	14.2	215	7.6	1.8	150	69.4	365	12.1	1.4
(b) Non-Hodgkin lymphoma	2	1.2	37	5.2	70	7.4	82	8.3	191	6.8	3.2	38	17.6	229	7.6	2.7
(c) Burkitt lymphoma	0	0	22	3.1	26	2.8	38	3.8	86	3.1	3.5	5	2.3	91	3.0	3.8
(d) Miscellaneous lymphoreticular neoplasms	7	4.1	4	0.6	3	0.3	4	0.4	18	0.6	2.0	1	0.5	19	0.6	1.7
(e) Unspecified lymphomas	0	0	3	0.4	8	0.8	5	0.5	16	0.6	4.3	0	0	16	0.5	4.3
III Central nervous system tumors	50	29.3	294	41.5	378	40.2	309	31.2	1031	36.7	1.2	81	37.5	1112	36.7	1.2
(a) Ependymomas and choroid plexus tumors	6	3.5	52	7.3	31	3.3	22	2.2	111	3.9	0.8	9	4.2	120	4.0	0.8
(b) Astrocytomas	19	11.1	112	15.8	130	13.8	121	12.2	382	13.6	1.3	28	13.0	410	13.5	1.4
(c) Intracranial and intraspinal embryonal neoplasms	13	7.6	55	7.8	81	8.6	45	4.5	194	6.9	1.7	7	3.2	201	6.6	1.8
(d) Other gliomas	0	0	10	1.4	16	1.7	15	1.5	41	1.5	1.2	8	3.7	49	1.6	1.2
(e) Other specified intracranial and intraspinal neoplasms	6	3.5	30	4.2	43	4.6	60	6.1	139	4.9	0.8	28	13.0	167	5.5	0.9
(f) Unspecified intracranial and intraspinal neoplasms	6	3.5	35	4.9	77	8.2	46	4.6	164	5.8	1.0	1	0.5	165	5.5	1.0
IV Neuroblastomas	116	68.0	156	22.0	44	4.7	17	1.7	333	11.8	1.3	6	2.8	339	11.2	1.2
(a) Neuroblastoma and ganglioneuroblastoma	116	68.0	156	22.0	44	4.7	12	1.2	328	11.7	1.2	4	1.8	332	11.0	1.2
V Retinoblastoma	39	22.9	57	8.0	6	0.6	1	0.1	103	3.7	1.6	0	0	103	3.4	1.6
VI Renal tumors	37	21.7	123	17.3	38	4.0	7	0.7	205	7.3	1.0	2	0.9	207	6.8	1.0
(a) Nephroblastoma and other nonepithelial renal tumors	36	21.1	123	17.3	36	3.8	6	0.6	201	7.1	1.0	0	0	201	6.6	1.0
VII Hepatic tumors	15	8.8	20	2.8	5	0.5	9	0.9	49	1.7	1.2	2	0.9	51	1.7	1.2
(a) Hepatoblastoma	14	8.2	19	2.7	0	0	3	0.3	36	1.3	1.4	0	0	36	1.2	1.4
(b) Hepatic carcinomas; (c) Unspecified malignant hepatic tumors	1	0.6	1	0.1	5	0.5	6	0.6	13	0.5	1.3	2	0.9	15	0.5	0.9
VIII Malignant bone tumors	3	1.8	10	1.4	62	6.6	161	16.3	236	8.5	1.0	34	15.7	270	8.9	1.0
(a) Ostosarcomas	0	0	1	0.1	31	3.3	96	9.7	128	4.6	0.9	20	9.3	148	4.9	1.1
(c) Ewing tumor and related sarcomas of the bone	0	0	8	1.1	23	2.4	49	4.9	80	2.8	1.2	11	5.1	91	3.0	1.2
(c) Other specified malignant bone tumors	2	1.2	0	0	3	0.3	5	0.5	10	0.4	1.0	0	0	10	0.3	1.0
(d) Unspecified malignant bone tumors	1	0.6	1	0.1	4	0.4	6	0.6	12	0.4	0.3	0	0	12	0.3	0.3
IX Soft tissue and other extraosseous sarcomas	22	12.9	74	10.4	67	7.1	79	8.0	242	8.6	1.2	37	17.1	279	9.2	1.3
(a)Rhabdomyosarcomas	9	5.3	54	7.6	35	3.7	22	2.2	120	4.3	1.1	9	4.2	129	4.3	1.2
(b), (c), (d), (e)^2^	13	7.6	20	2.8	32	3.4	57	5.7	122	4.3	1.3	28	13.0	150	5.0	1.3
X Germ cell tumors	25	14.7	30	4.2	23	2.4	35	3.5	113	4.0	0.7	56	25.9	169	5.6	1.3
(a) Intracranial and intraspinal germ cell tumors	6	3.5	1	0.1	8	0.8	10	1.1	25	0.9	1.1	2	0.9	27	0.9	1.1
(b) Malignant extracranial and extragonadal germ cell tumors	12	7.0	18	2.5	2	0.2	0	0	32	1.1	0.4	1	0.5	33	1.1	0.4
(c) Malignant gonadal germ cell tumors	7	4.1	11	1.6	10	1.1	24	2.4	52	1.9	0.9	49	22.7	101	3.3	2.3
XI Other malignant epithelial neoplasms and malignant melanomas	1	0.6	4	0.6	28	3.0	79	8.0	112	4.0	0.9	120	55.5	232	7.7	0.9
(a) Thyroid carcinomas	0	0	1	0.1	10	1.1	34	3.4	45	1.6	0.6	59	27.3	104	3.4	0.6
(b) Malignant melanomas	0	0	0	0	6	0.6	11	1.1	17	0.6	0.7	31	14.3	48	1.6	0.7
XII Other and unspecified malignant neoplasms	4	2.3	12	1.7	2	0.2	4	0.4	22	0.8	1.2	1	0.5	23	0.8	1.1
All embryonal tumors	225	132.0	448	63.2	189	20.1	82	8.3	944	33.6	1.3	17	7.9	961	31.7	1.3
All non-embryonal tumors	156	91.5	1070	150.9	1050	111.5	1191	120.3	3467	123.3	1.2	592	273.8	4059	134.1	1.2
All tumor types	381	223.4	1518	214.1	1239	131.6	1273	128.5	4411	156.9	1.3	609	281.7	5020	165.8	1.3

^1^The period 1970–1975 is restricted to the Province of Torino. Data for adolescents (15–19) are available from 2000. Myelodysplasias are available from 2000 (n = 13).

^2^The composite category includes cases from the following subgroups: ICCC IXb Fibrosarcomas, peripheral nerve sheath tumors, and other fibrous neoplasms, IXc Kaposi sarcoma, IXd Other specified soft tissue sarcomas and IXe Unspecified soft tissue sarcomas

**Table 3:** Childhood Cancer Registry of Piedmont 1976–2011: Number of cases (n) and annual percent change (APC) with corresponding 95% confidence interval.

Childhood cancer registry of Piedmont 1976–2011^1^	1976–2011		2000–2011^2^			
International Classification for Childhood Cancer, Third edition	*0–14 yrs*		*0-14yrs*		*15–19*	
	*n*	*APC (95%CI)*	*n*	*APC (95%CI)*	*n*	*APC (95%CI)*
I Leukemias	1168	**0.6 (0.0; 1.2)**	378	-0.6 (-5.4; 4.4)	76	2.0 (-5.3; 9.9)
(a) Lymphoid leukemia	931	**0.9 (0.3; 1.4)**	306	-0.2 (-4.7; 4.5)	36	7.1 (-0.7; 15.7)
(b) Acute myeloid leukemia	180	-0.7 (-4.7; 3.5)	46	2.1 (-35.3; 61.2)	27	2.2 (-6.4; 11.4)
II Lymphomas	449	**1.7 (0.6; 2.7) (a)**	151	-1.4 (-6.8; 4.3)	194	-1.7 (-5.6; 2.3)
		-12.2 (-33.2; 15.4)				
(a) Hodgkin lymphoma	182	2.2 (-1.7; 6.3)	70	-2.3 (-9.1; 5.0)	150	-0.5 (-4.9; 4.1)
(b) Non-Hodgkin lymphoma	156	-1.0 (-5.0; 3.2)	43	39.6 (-20.3; 144.6)	38	-3.2 (-13.1; 7.8)
III CNS tumors^3^	728	**1.8 (0.9; 2.7)**	323	-0.3 (-4.5; 4.2)	81	4.6 (-3.0; 12.9)
(a) Ependymomas and choroid plexus tumors	90	4.8 (-6.1; 16.9)	41	-4.5 (-13.0; 4.9)	9	(b)
(b) Astrocytomas	288	1.2 (-0.4; 2.8)	122	-3.0 (-8.7; 3.1)	28	5.6 (-5.4; 18.7)
(c) Intracranial and intraspinal embryonal neoplasms	131	**1.7 (0.1; 3.4)**	55	3.8 (-4.8; 13.1)	7	(b)
IV Neuroblastomas	286	**1.2 (0.2; 2.1)**	114	-2.4 (-7.0; 2.5)	6	(b)
(a) Neuroblastoma and ganglioneuroblastoma	282	**1.1 (0.2; 2.1)**	113	-2.5 (-7.3; 2.6)	4	(b)
V Retinoblastoma	81	0.7 (-11.1; 14.2)	24	-9.9 (-49.5; 60.5)	0	(b)
VI Renal tumors	167	0.2 (-1.1; 1.5)	54	-4.5 (-10.7; 2.2)	2	(b)
(a) Nephroblastoma and other nonepithelial renal tumors	164	0.2 (-1.1; 1.5)	53	-4.5 (-10.6; 2.2)	0	(b)
VII Hepatic tumors	42	3.8 (-13.4; 24.4)	**17**	**195.9 (6.6; 721.7)**	2	(b)
(a) Hepatoblastoma	30	8.1 (-13.4; 34.9)	13	209.0 (-1.2; 866.5)	0	(b)
VIII Malignant bone tumors	190	0.0 (-4.1; 4.4)	51	-4.1 (-14.8; 7.9)	34	9.2 (-1.9; 21.4)
(c) Ewing tumor and related sarcomas of the bone	74	-5.9 (-18.9; 9.1)	20	-7.6 (-75.0; 242.1)	11	(b)
IX Soft tissue and other extraosseous sarcomas	206	0.5 (-0.7; 1.7)	64	-4.8 (-12.2; 3.2)	37	-2.3 (-12.0; 8.6)
(a)Rhabdomyosarcomas	144	-1.9 (-8.3; 4.9)	33	6.6 (-52.2; 137.7)	9	(b)
X Germ cell tumors	94	2.4 (-5.9; 11.5)	35	-25.5 (-56.3; 27.1)	56	-3.1 (-9.7; 4.1)
(a), (b), (c)	91	2.7 (-5.8; 11.9)	35	-25.5 (-56.3; 27.1)	52	-2.6 (-9.0; 4.2)
XI Other malignant epithelial neoplasms and malignant melanoma	88	3.9 (-1.4; 9.5)	41	27.9 (-20.4; 105.8)	120	2.4 (-3.2; 8.3)
(a) Thyroid carcinomas	37	20.4 (-2.3; 48.3)	17	125.8 (-23.6; 567.5)	59	3.7 (-6.3; 14.8)
(b) Malignant melanomas	12	14.3 (-8.0; 42.1)	5	(b)	31	-1.1 (-13.5; 13.1)
XII Other and unspecified malignant neoplasms	6	(b)	2	(b)	1	(b)
All embryonal tumors	801	**1.1 (0.5; 1.6)**	289	-0.6 (-3.6; 2.6)	17	-4.5 (-15.8; 8.3)
All non-embryonal tumors	2857	**1.2 (0.7; 1.6)**	965	-1.0 (-4.0; 2.1)	592	0.3 (-2.3; 2.9)
All tumor types	3658	**1.1 (0.8; 1.5)**	1254	-0.9 (-3.4; 1.7)	609	0.9 (-1.8; 3.5)

^1^Data for adolescents (15–19) are available from 2000. Myelodysplasias are available from 2000 (n = 13).

^2^APC for the period 2000–2011 was calculated including myelodysplasias.

^3^ Number of cases and the APCs for CNS tumors were estimated from 1982–2011

(a) Breakpoint in 2007

(b) APC not estimated because of insufficient number of cases

Leukemia is the most common cancer type in children. Acute lymphoid leukemia (ALL) accounts for around 73% of all leukemia cases. ALL incidence peaks in the 1–4 year age group, with a rate of 75.9 (69.6; 82.6) in the whole observation period (1967–2011) (Table 2, Fig 2). We found a statistically significant increase in all leukemias incidence rates in the period 1976–2011, APC 0.6% (0.0; 1.2), which seems mainly driven by the increase of ALL, APC 0.9% (0.3; 1.4) (Table 3).

Lymphomas are the most common cancer type in adolescents. Their incidence rates in children are 18.7 (17.1–20.4) and 89.7 (77.6–103.3) in adolescents (Table 2). Male predominance is remarkable in both Hodgkin lymphoma (HL) and Non-Hodgkin lymphoma (NHL) (Table 2). HL incidence rate among adolescents is particularly high, 69.4 (58.7–81.4) (Table 2). In children, the overall (1976–2011) time trend for all lymphomas exhibited a breakpoint, with increasing incidence until 2007, APC 1.7% (0.6; 2.7), followed by a non statistically significant decline afterwards (Table 3).

CNS neoplasms are the second most common cancer type in children (23.4% of all tumors) and third most common cancer type in adolescents (13.3% of all tumors), with high rates in both children 36.7 (34.5–39.0) and adolescents 37.5 (29.8–46.6) (Table 1, Fig 2). In the chosen observation period (from 1982, to account for improved diagnostic techniques, to 2011) CNS tumors exhibited a significant increase in incidence rates, APC 1.8% (0.9; 2.7) (Table 3). In the analysis by tumor grading, CNS tumors with both grade I and IV showed a strong increase, APC 3.6% (2.5; 4.8) and 2.4% (0.7; 4.2), respectively. On the contrary, grade II and III tumors (grouped together) showed an increase until 1997 APC 5.1% (0.4; 10.0) followed by a decline APC -6.4% (-11.2; -1.5). (data not shown).

The incidence rate of neuroblastoma is highest in children in their first year of life, 68.0 (56.2–81.6) (Table 2). The trend analysis showed an increase in incidence rates for the overall period (1976–2011) in all children APC 1.2% (0.2; 2.1) (Table 3) and in those in their first year of life APC 2.5% (0.7; 4.2). When we restricted the neuroblastoma trend analysis to the period 1990 to 2011, increases in time trends lost statistical significance: APC 1.7% (-0.4;3.7) for all children, and APC 2.0% (-1.5; 5.7) for <1 year old children (data not shown).

The analysis of the period 2000–2011 (with the exception of hepatoblastomas in children) showed no statistically significant variation in incidence rates, neither in children, nor in adolescents. The major ICCC groups (namely leukemias, lymphomas, CNS tumors and neuroblastomas) that showed an increase for the 1976-2011period in children, show decreasing rates in 2000–2011 (Table 3).

## Discussion

Analyzing the overall time trends (1976–2011) in children, we found statistically significant increases in incidence rates in all tumors types combined, and in both embryonal and non-embryonal tumors when analysed as separate categories. In the analysis by ICCC diagnostic groups, we found increases in leukemias (mainly due to the increase of ALL), lymphomas, CNS tumors (from 1982) and neuroblastomas.

The analysis of the most recent period (2000–2011) did not provide sufficient evidence to claim that the increasing trends that have characterized the whole time series have now started inverting their tendency, since the rarity of cancer in the age groups considered is affected by large variability that hampers interpretation.

When we interpret our findings, we must consider that changes in data collection, classification or changes in diagnostic procedures might have influenced our results.

The quality and completeness of data collected from CCRP has been high and its data have been included in all major studies on childhood cancer incidence in Italy and Europe [4,5,12,23]. In the first 10 years of registration (1967–1976) there were 55 cases identified only through death certificate (DCO), but only one after 1976 (Table 1). The proportion of microscopic verification (MV) increased until the last period, where it showed a slight decline mirrored by an increase in the percentage of diagnoses exclusively based on imaging, showing increased diagnostic capacity rather than a decline in the quality of registration (Table 1). After 1975, around 95% of all cases have histological confirmation.

In our observation window, several relevant diagnostic changes occurred. From mid 1970’s the distinction between ALL and acute myeloid leukemia (AML) became reliable through the introduction of flow cytometry so that the proportion of unspecified leukemias became negligible. For CNS tumors, major diagnostic improvements included the introduction of CT scans and their widespread use in the 1980’s, followed by that of magnetic resonance in the 1990’s, and of cranial ultrasound in newborns from the 1990’s. Relevant diagnostic changes for neuroblastoma (and possibly for other embryonal abdominal neoplasms, such as nephroblastoma and hepatoblastoma) were the antenatal and perinanatal ultrasound imaging in mid 1980’s and the widespread use of hip ultrasound from mid 1990’s.

It is unlikely that improved classification explains the observed ALL increasing trends. In our data, from mid 1970’s, the proportion of unspecified leukemias has been constantly low, and that of MV cases for ALL was 100%. Increasing leukemia time trends have been observed in previous CCRP [6,24] and Italian [4,5] studies. Incidence rates in Italy mostly increased in the 1980’s until mid 1990’s, especially those of ALLs in the 1–4 age group. A similar behavior was observed in other European countries [1,2,25–28], more pronouncedly in western then eastern Europe. SEER data in a similar period (1975–2013) also show an increase in leukemia rates (APC 0.7%, p<0.05) [3].

HL behavior is particularly interesting because Italian rates among children and adolescents (0–19 years old) are the highest of the world [29]. In USA [30] and Nordic countries [31], rates were approximately half of those observed in Piedmont and in Italy [5]. (Table 4) Our analysis did not show statistically significant trends for HL, but showed an increase in all lymphomas considered together in 0–14 years old up to 2007, followed by a decrease. APC was higher for HL than for all lymphomas (2.2 vs 1.7). A previous CCRP study [6] for the period 1967–2001, found a statistically significant increased incidence rates for HL in 10–14 year old children.

**Table 4:** Hodgkin lymphoma rates by age group and gender in Piedmont, Italy, Nordic countries and the USA.

	0–4 yrs		5–9 yrs		10–14 yrs		15–19 yrs	
	*Boys*	*Girls*	*Boys*	*Girls*	*Boys*	*Girls*	*Boys*	*Girls*
CCRP (2000–2011)	3.5	0	10.9	2.9	23.9	24.3	66.4	72.6
AIRTUM (2003–2008)			7.7	2.9	25.0	27.8	58.3	71.3
NORDCAN (2000–2013)	1.0	0	5.0	1.0	13.0	12.0	34.0	40.0
SEER^1^ (2000–2013)	0.6	0.4	5.8	2.6	12.4	12.1	34.8	35.6

CCRP = Childhood Cancer Registry of Piedmont; AIRTUM = Italian Association of Cancer Registries; NORDCAN = Association of the Nordic Cancer Registries; SEER = Surveillance, Epidemiology, and End Results Program

^1^ The rates from SEER refer to numbers from 9 SEER registries, race: white.

The AIRTUM working group [5] found an increase in children for all lymphomas up to 1999 and a decline afterwards. In Europe, for the period 1975–1997, the increase in HL was found in both the 10–14 (AAPC (average annual percent change) 1%) and the 15–19 (AAPC 3.5%) age groups[32]. In USA, HL showed a decline (1975–2013, 0–19 years, APC -0.6%, p<0.05) and NHL an increase (1975–2013, 0–19 years, APC1.1%, p<0.05) [3].

There were previous reports of increasing trends of CNS tumors in Europe and USA [33–35]. The Automated Childhood Cancer Information System study on childhood CNS tumors [33] reported increased incidence in Europe in the period 1978–1997, that the authors partly explained with improved diagnostic methods and changes in classification and registration. In Italy, the AIRTUM study [5] found increasing trends in all CNS tumors considered together, but the analysis restricted to those with malignant behavior showed stable rates. In Piedmont, a previous study analyzing trends from 1967–2001 [6] found significantly increasing CNS tumor trends that showed a constant gradual change rather than a step-increase that usually follows the introduction of new diagnostic techniques. In our analysis, we found statistically significant increases in incidence rates of CNS tumors even after starting the analysis from 1982 to account for the introduction of CT scans. When we looked into the incidence pattern of CNS by WHO grade, we found that grade I tumors that are more likely to be affected by improvements in diagnostics, have indeed been increasing. However, also the incidence of highly malignant grade IV tumors has been increasing. Given the rapidly growing and aggressive nature of these tumors, it is unlikely that a diagnostic anticipation may translate into an apparent increasing trend, thus leading us to the conclusion that the increasing trends of CNS tumors might reflect a true change in the risk factors patterns of exposure.

In Europe, the incidence of embryonal tumors like neuroblastoma, nephroblastoma and, to a lesser extent, retinoblastoma and hepatoblastoma, seem to have increased [1,11,36–39]. The significant increases for all embryonal tumors for the overall period, are mostly due to the increase in rates of cases diagnosed in the first year of life. This increase might be partly due to introduction of antenatal and perinanatal ultrasound imaging in Italy in the mid 1980’s and the use of hip ultrasound from around 1995, especially for tumors that in a small proportion may regress spontaneously, such as neuroblastoma. Indeed, when the trend analysis was restricted to the period 1990–2011, increases in time trends for neuroblastoma persisted but lost statistical significance and time trends from 2000 show decreasing rates for embryonal tumors in children, including neuroblastoma and retinoblastoma. Hepatoblastomas, however, showed an increase, although our analysis is based on small numbers.

In conclusion, in Piedmont, the analysis of trends over a long period (1976–2011) shows that the incidence of most tumor types has increased. When we looked more closely at the most recent years of observation (2000–2011), we found that rates seem to have started levelling off. However, large variability hampers interpretation of trends pattern in short periods. In Europe, the upward trends were detected more then 12 years ago, but as yet no satisfying explanation for the past increases has been found. Efforts must be made to understand and interpret this peculiar and still ununderstood pattern of incidence of childhood cancer.
